# Towards PCB-Based Miniaturized Thermocyclers for DNA Amplification

**DOI:** 10.3390/mi11030258

**Published:** 2020-02-28

**Authors:** Georgia D. Kaprou, Vasileios Papadopoulos, Christos-Moritz Loukas, George Kokkoris, Angeliki Tserepi

**Affiliations:** Institute of Nanoscience and Nanotechnology, National Center for Scientific Research “Demokritos”, Patr. Gregoriou E’ and 27 Neapoleos str., 15341 Aghia Paraskevi, Attiki, Greece; gkap@unileon.es (G.D.K.); v.papadopoulos@inn.demokritos.gr (V.P.);

**Keywords:** printed circuit board (PCB), microfluidics, micro polymerase chain reaction (microPCR), static, multi-well PCR, simulation, genomic *Salmonella* DNA

## Abstract

In recent years, printed circuit board (PCB)-based microfluidics have been explored as a means to achieve standardization, seamless integration, and large-scale manufacturing of microfluidics, thus paving the way for widespread commercialization of developed prototypes. In this work, static micro polymerase chain reaction (microPCR) devices comprising resistive microheaters integrated on PCBs are introduced as miniaturized thermocyclers for efficient DNA amplification. Their performance is compared to that of conventional thermocyclers, in terms of amplification efficiency, power consumption and duration. Exhibiting similar efficiency to conventional thermocyclers, PCB-based miniaturized thermocycling achieves faster DNA amplification, with significantly smaller power consumption. Simulations guide the design of such devices and propose means for further improvement of their performance.

## 1. Introduction

The interest of both academia and industry in microfluidic devices has been continuously growing for the last three decades, thanks to their advantages, including the capability of handling very small quantities of expensive reagents and scarce samples, the performance of high resolution, precise and sensitive detection, and the reduction in analysis time, cost, and footprint [1]. However, the long-awaited widespread penetration of microfluidics into the market has still not been achieved, to a large extent due to factors such as reduced compatibility with mass manufacturing, lack of standardization, and still existing system integration issues.

In recent years, several approaches have been developed and a few initiatives have been undertaken to eliminate most of these issues, reduce the communication gap between academia and industry, and ultimately improve the commercialization potential of microfluidics. Examples include the initiative for microfluidics standardization (MicroFluidicsManufacturing, the European initiative for the standardization and manufacturability of complex micro-fluidic (MF) devices [2]) and the establishment of a large consortium of major industrial and academic partners to provide guidelines for seamless integration of microfluidic components with sensors and actuators. Members of this consortium also introduced [3,4] the concept of the fluidic circuit board (FCB), a standardized modular platform, assembling easy-to-fabricate microfluidic building blocks compatible with mass manufacturing and comprising fluidic as well as electrical connections. An independent approach, the lab-on-printed circuit board (Lab-on-PCB), while suggested many years ago for the fabrication of microfluidics [5], has recently re-emerged as a very strong candidate [6], owing to its inherent upscaling potential: the PCB industry, although now focused on consumer electronics, is well-established all around the world, with standardized fabrication facilities and processes. As already demonstrated in several works, the Lab-on-PCB approach enables seamless integration of microfluidics, sensors, and electronics [7,8,9,10,11,12] and promises commercial upscalability, low cost, and standardization of microfluidics. Owing to these characteristics, Lab-on-PCB devices can easily be upscaled, provided more processes and prototypes adapted to the PCB industry are proposed.

Recent examples include the introduction of fully PCB-compatible microfluidic devices integrated with microheaters for cell lysis, DNA extraction, and amplification [13,14], as well as for quantitative Polymerase Chain Reaction (qPCR) with integrated, PCB-based electrochemical biosensors and thermal cyclers [15]. In addition, processes have been developed, adapted to the PCB industry, not only for the patterning but also the sealing [16] of extremely demanding microdevices for rapid DNA amplification and with low power consumption [17], as well as the implementation of commercially fabricated PCB-based electrochemical biosensors in DNA diagnostic microsystems [18]. Furthermore, devices for post-amplification processes, such as enzymatic DNA digestion, have been introduced [19,20], while processes have been developed to functionalize PCB-relevant materials and improve the performance of PCB-based microfluidic devices [21,22].

The progress achieved or being under development in recent years on PCB-based diagnostic microsystems certainly paves the way for the commercialization of nucleic acid-based diagnostic tests for infectious diseases [23,24], so far dominated by PCR performed in conventional thermocyclers. However, conventional PCR machines are expensive and not portable, thus posing barriers to PCR adoption in field or point-of-care (PoC) applications. Despite the fact that the first commercial PCB-based chip for nucleic acid testing (i.e., the TrueNAT^®^ chip) carried out in a portable real-time PCR platform has been developed by Bigtec Labs and commercialized by Molbio Diagnostics [25], the chip (proprietary technology) can handle and process only one sample at a time [24]. In this work, PCB is evaluated, for the first time to the best of our knowledge, as a platform for the implementation of static microPCR, focusing on its conditional capability of performing multi-well reactions, in addition to its low power consumption, compared to conventional thermocyclers (based on Peltier elements) [26]. The presented static microPCR is demonstrated to exhibit comparable DNA amplification efficiency at the expense of a much smaller power, while further improvements of its performance (duration, energy consumption, and temperature uniformity) are anticipated following the guidelines dictated by detailed numerical calculations.

## 2. Materials and Fabrication, Methods, and Calculations

The entire microPCR device consisted of a chip comprising microfluidic chambers and a resistive microheater, all integrated on PCB, and a temperature controller for thermocycling of the chip and sample.

### 2.1. Printed Circuit Board (PCB) Microheaters

**Figure 1:**
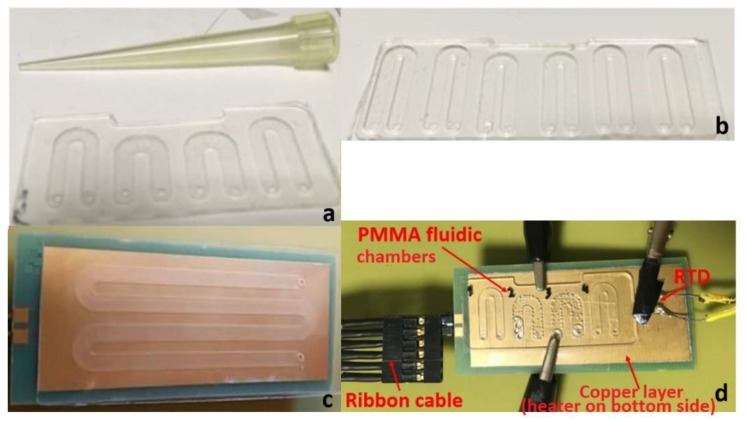
(**a**) Poly(methyl methacrylate) PMMA fluidic chip with 4 u-shaped chambers; (**b**) PMMA fluidic with 6 u-shaped chambers; (**c**) PMMA fluidic chip on top of a thin printed circuit board (PCB) microheater with an external temperature-homogenizing copper layer; (**d**) Experimental set-up for temperature measurements during thermocycling of a static micro polymerase chain reaction (microPCR) chip.

Resistive microheaters were designed for integration in the static microPCR device. An open source software, Kicad (https://kicad-pcb.org/ (Online) (Visited on 27 February 2020)), was used for designing the microheaters as meandering copper tracks in order to provide sufficient length and thus achieving the desired resistance value. The microheaters were mass-fabricated on commercially available PCB substrates by a major PCB vendor (Eurocircuits LTD, Mechelen, Belgium) according to our specifications included in the CAD designs. The thickness of the PCB substrate was 1.6 and 0.8 mm for the thick and the thin microheater, respectively. The area covered by the thin microheater (3 cm × 6 cm) was 1.5 times larger than that of the thick (2.5 cm × 5 cm) so that the PCB area was compatible with six and four fluidic chambers, respectively (see Figure 1a,b). Each microheater could accommodate multiple layers of copper. The thickness of the internal copper layers was 18 μm, whereas the external thickness was 12 μm. The copper track width (used for the microheaters) was 100 μm. In the case of the thick microheater, a four-copper layer PCB was employed with the microheater patterned solely on one internal copper layer, which led to a resistance value of 12.5 Ohm. In the case of the thin microheater, a two-copper layer PCB was employed, with the resistive microheater patterned on one of the external copper layers reaching a resistance value of 23 Ohm, whereas the second one was used as a solid copper surface to improve the temperature uniformity across the heated area. The temperature coefficient of resistance for the copper track was determined in a previous work to be 0.0036 ± 0.0002 °C^−1^ [27].

### 2.2. Poly(methyl Methacrylate) PMMA Microfluidics

Computer numerical control (CNC) (LPKF Laser & Electronics AG, Osteriede 7, D-30827 Garbsen, Germany) machining was used in order to pattern the microfluidic network on 1 mm thick Poly(methyl methacrylate) (PMMA) substrates. First, the desired microfluidic network was designed using commercial software (i.e., Corel Draw^®^, Autocad^®^, Corel Corporation, Ottawa, ON, Canada, (https://www.autodesk.com/education/free-software/autocad (Online) (Visited on 27 February 2020)), and then the design was imported into the specialized software CircuitPro utilized by the LPKF (LPKF Laser & Electronics AG, Osteriede 7, D-30827 Garbsen, Germany). Good adherence of the PMMA substrate to the milling table was ensured by vacuum. CNC machining also offered the possibility to drill through holes for the inlet and outlet. Subsequently, the patterned fluidics were cleaned with propanol and water and dried with pressurized air, which also assisted the removal of any milling residues. The sealing of the microdevices was performed using Clear Polyolefin StarSeal (STARLAB (UK), Ltd 5 Tanners Drive, Blakelands, Milton Keynes MK14 5BU, United Kingdom) (PCR compatible) from StarLab. This product is typically used for covering multi-well plates and is PCR compatible. The temperatures it withstands range from −70 °C to 110 °C. It consists of a 0.05 mm polyolefin film, single-coated with a pressure sensitive acrylate adhesive. The product can be applied by hand or using a laminator (at 90 °C, optimally).

The microPCR chip (see Figure 1) comprises many (typically four to six) u-shaped chambers in order to simultaneously perform multiple amplification reactions at a single thermocycling run. The width of the microchambers was 2 mm and the depth varied from 0.3 mm to 0.5 mm. The total length of the microchambers was between 25 and 30 mm, thus leading to an approximate total chamber volume of 25 μL. Good thermal conductivity between the PMMA microfluidic chip and the PCB microheater was achieved by using a thermally conductive paste.

### 2.3. Temperature Verification via Resistance Temperature Detectors

For a successful two-temperature (2T) PCR, the temperatures achieved by means of the microheaters and their control as well as the ramping rates were investigated independently from the microheater resistance measurements. As an external reference for sensing the temperature, a platinum resistance (Pt100 surface element) temperature detector (RTD) (OMEGA Engineering, Manchester, UK) was used. Figure 1d illustrates the set-up used, while running DNA amplification, for measuring the actual temperatures on a thin microheater chip bearing an external copper layer for improved temperature uniformity.

### 2.4. Biological Protocols

The KAPA2G Fast ReadyMix (KapaBiosystems) kit was used according to supplier’s instructions. Each primer was used at a final concentration of 10 μM. Purified genomic *Salmonella* DNA was used as template. Two pairs of primers based on the STM4497 gene were selected for the amplification of a 117 bp and a 228 bp target gene fragment because of their high specificity, stability, and good sensitivity [28]. The sets of the primers used in these experiments for the amplicons corresponding to 117 bp and the 228 bp were for the forward primer 5′- CAACACCTGCAGGATAATCCAATATTATTAAG (32 bp, *T_m_* = 64.6 °C), for the reverse primer 5′- CTGTTATTTCCTGCGTGGATATTTCTTTAG (30 bp, *T_m_* = 64.2 °C), the forward primer 5′- GGATCACTAAGCTGTGGATTACCTATTATC (30 bp, *T_m_* = 64.4 °C), and for the reverse primer 5′- TATTCAGCGTAAAGAAGATTAACAGCAATAAG (32 bp, *T_m_* = 64.7 °C), respectively. Amplification experiments were carried out in a conventional thermocycler (i-cycler from Biorad™, Hercules, CA, USA) and in static microPCR devices.

### 2.5. The Modeling Framework

**Figure 2:**
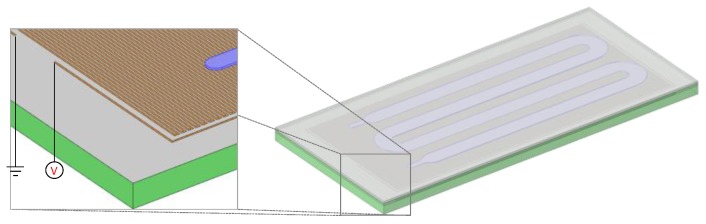
Schematic of the microPCR design used for the computational study. The PCB (green) layer incorporated a meander-shaped copper line (brown) operating as a microheater. A PMMA layer (gray) lay on top of the PCB layer and included a microchannel which was sealed by a polyolefin layer.

The objective of the computational study was the investigation of means to improve the performance of PCB-based microPCR in terms of duration, energy consumption, and temperature uniformity for a two-temperature (65 °C and 95 °C) PCR protocol. The geometry of the microPCR used in the computational study is shown in Figure 2. It included a PCB layer (with thickness of 1.68 mm) with an embedded microheater, i.e., a copper line. The thickness, width, and total length of the copper line were 25 μm, 100 μm, and 4.973 m. On top of the PCB layer, there was a PMMA layer (with thickness of 300 μm) with meander-shaped microchannel which was sealed with a polyolefin layer (with thickness of 50 μm). The depth, width, and total volume of the microchannel were 100 μm, 2.5 mm, and 41 μL, respectively. The footprint of the microPCR design was 54.1 × 24.9 mm^2^. The footprint as well as the stack of materials in the PCB layer were very close to the thin chip used in the experiments (cf. Section 2.1).

The study was based on a modeling framework [19], including an energy balance in the solid domains and the fluid in the microchannel, which read:

(1) $$\rho C_{p}\frac{dT}{dt}=\nabla \cdot \left(k\nabla T\right)+Q,$$

where *T*, *k*, *ρ*, and *C_p_* are the temperature, the thermal conductivity, the density, and the heat capacity of the solid or the fluid. *Q* is the heat generation rate at the microheater. It was zero for all domains except for the microheater.

The Joule heating mechanism and the details of the geometry of the microheaters (operating as resistances) were taken into account. The heat generation rate according to the Joule heating read:

(2) Q=J⋅E.

*E* is the electric field in the microheater and *J* is the current density, which read:

(3) J=σE,

and was calculated by the current conservation equation, i.e.,

(4) ∇⋅J=0.

*σ* is the electrical conductivity of the microheater, which, for the case of copper, was linear, with the following formula

(5) $$\sigma =\frac{1}{\rho_{0}\left[1+\alpha (T-T_{0})\right]},$$

where *ρ_0_* is the electrical resistivity at temperature equal to *T*_0_, and *a* is the temperature coefficient of resistivity.

Heat losses by convection and radiation were applied on all external surfaces of the device. The heat transfer coefficient is a function of the surface temperature, the latter coming from a computational study for the heat losses of microfluidic devices [29]. A time varying voltage was applied across the microheater in order to achieve the desired thermal cycle, resembling the functionality of a simplified temperature controller. During heating, a constant voltage was applied, during cooling the temperature controller was switched off. Finally, electrical insulation was applied to all other boundaries of the heaters.

The numerical calculations required for the study were performed by the finite element method implemented with the commercial code COMSOL (COMSOL Inc., Stockholm, Sweden).

## 3. Results and Discussion

### 3.1. Experimental

For the validation of the static microPCR, an optimized 2T protocol was developed for efficient and fast DNA amplification based on PCR. Different *Salmonella* genomic DNA concentrations were used, ranging from 1.25 ng (2.5 × 10⁵ copies) down to 0.00125 ng (250 copies) per 25 μL PCR reaction, and their amplification was carried out in microPCR chips on thermocycled PCB substrates. The results are presented and discussed in the following subsections.

#### 3.1.1. Optimization of a Two-Temperature Polymerase Chain Reaction (PCR) Protocol

**Figure 3:**
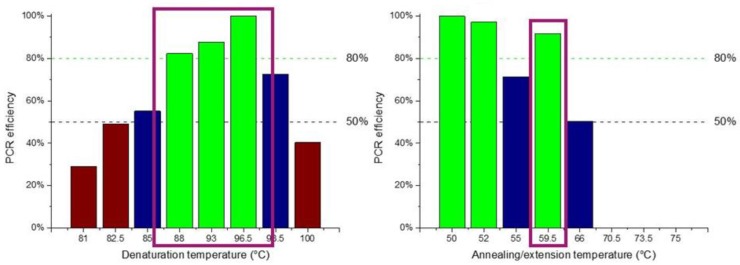
PCR temperatures for optimal amplification efficiency following a two-temperature (2T) protocol. The bars represent band intensities on the gel normalized with the most intensive one (100%). Green, blue, and brown bars indicate temperature ranges achieving amplification efficiency higher than 80%, between 50 and 80%, and lower than 50%, respectively.

In order to optimize a 2T PCR protocol, several reactions were run on a conventional thermocycler to find the optimal temperature ranges for enhanced amplification efficiency. The DNA template used was purified genomic *Salmonella* DNA. Two sets of experiments were performed: (i) gradient temperature for the denaturation step with temperatures ranging from 81 °C to 100 °C in steps of 3–5 °C and constant temperature for the annealing/extension step set at 60 °C, and (ii) constant temperature for the denaturation step at 95 °C and gradient temperature for the annealing/extension step ranging from 50 °C to 70 °C. The duration of each step was 10 s and 30 s for the denaturation and annealing/extension steps, respectively, while 40 such cycles were performed. The results obtained are illustrated in Figure 3. The optimum temperatures for denaturation proved to be in the range of 88 °C to 97 °C, offering an efficiency higher than 80%, whereas for the annealing/denaturation 50–52 °C and 60 °C offered an efficiency higher than 90%.

Based on these results and targeting amplification as fast as possible, it was decided to perform the 2T PCR at 90 °C (for denaturation) and 60 °C (for annealing/extension) for minimum temperature difference, and employ 40 cycles for increased (>80%) amplification efficiency. By applying this protocol, good specificity for the desired product was expected as well as a rapid amplification, at the expense of some efficiency loss (due to the chosen denaturation temperature), which is not a significant compromise.

#### 3.1.2. Thermocycling with a Thick PCB Microheater Chip

Evaluation of the static microPCR using a thick PCB microheater was performed using two different amplicons and various purified genomic *Salmonella* DNA concentrations. The *Salmonella* genomic DNA concentrations used ranged from 1.25 ng (2.5 × 10⁵ copies) to 0.00125 ng (250 copies) per 25 μL PCR reaction.

**Figure 4:**
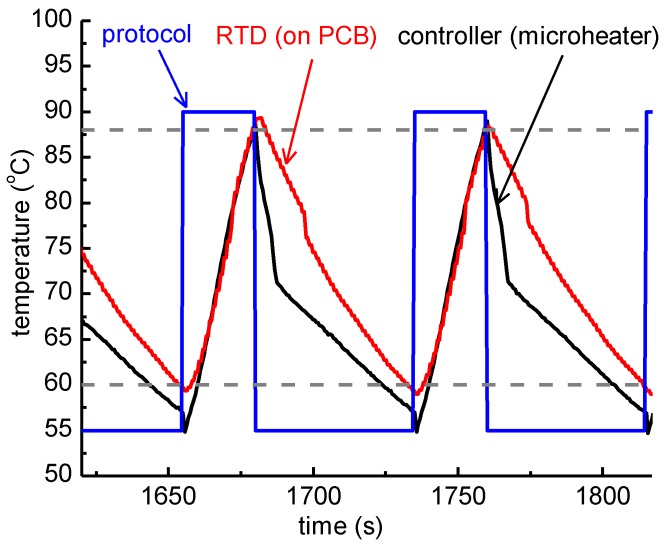
Graphical representation of the temperature measured while running an actual 2T thermocycling experiment with a thick PCB microheater chip. The protocol followed by the temperature controller was 25 s at 90 °C followed by 55 s at 55 °C (blue line). The temperature of the microheaters, as obtained from their resistance values acquired by the temperature controller, and the temperature recorded by the resistance temperature detector (RTD), are also shown. The dashed lines correspond to 88 °C and 60 °C accordingly.

The nominal temperature protocol applied to the temperature controller of the microheaters was 25 s at 90 °C (denaturation step) and 55 s at 55 °C (extension/annealing step). The duration of each step does not represent the actual residence time at each temperature step, since it also includes the time needed for the microheater to reach the set-point temperature, as shown in Figure 4. In fact, Figure 4 includes the nominal temperature profile for a 2T thermocycling experiment, as dictated by the protocol, as well as the temperature of the microheaters, as obtained from their resistance values acquired by the temperature controller, and the temperature as recorded by the RTD, which was in good thermal contact with the PCB where the PMMA microfluidic chip lay. In Figure 4, the temperature range for efficient amplification (>80%, according to the results shown in Section 3.1.1), i.e., above 88 °C and below 60 °C, is also noted.

According to the protocol followed, the residence time based on the RTD measurement, i.e., on the PCB, was 4.5 s for the denaturation step (temperature over 88 °C) and 3.7 s for the step of annealing/extension (temperature below 60 °C) (Table 1). The former is very close to the residence time based on the controller measurement, i.e., at the microheater (3.3 s), whereas the residence time for the annealing/extension step was 4.2-fold higher at the microheater (15.5 s).

Regarding the observed ramping rates, the cooling rate was estimated to be 0.5 °C/s and 0.6 °C/s from the RTD and the controller measurements, respectively. Accordingly, the heating rates were 1.1 °C/s and 1.4 °C/s from the RTD and the controller measurements, respectively. Both heating and cooling rates were slightly faster in the measurement from the temperature controller. The low (passive, as no external source is utilized) cooling rates can be attributed to the relatively high thickness of the PCB substrate (1.6 mm).

**Figure 5:**
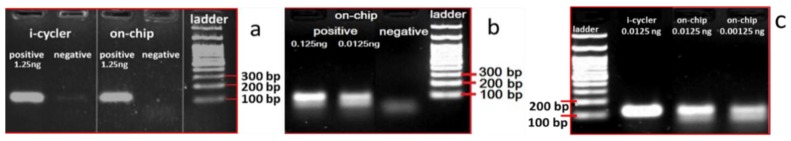
Agarose gel electrophoresis images depicting the (117 bp) products from the conventional thermocycler and the on-chip amplification for purified genomic *Salmonella* DNA using 4 different DNA concentrations, from 1.25 ng (**a**), to 0.125 and 0.0125 ng (**b**), down to 0.00125 ng (**c**), per 25 μL PCR reaction. The on-chip experiments were performed one at a time, in a microfluidic chamber lying in the central area of the fluidic chip, in thermal contact with a thick PCB microheater chip.

**Figure 6:**
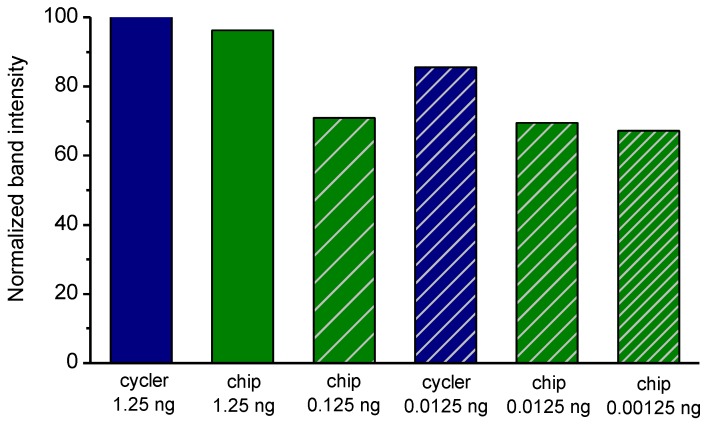
Comparison of normalized (to the intensity of the cycler with 1.25 ng DNA template) band intensities for PCR reactions of different DNA template amounts in the standard thermocycler (i-cycler) and the static microPCR after 40 cycles of the 2T PCR.

In Figure 5, images of the gel electrophoresis are presented for DNA (of various initial concentrations) amplified in both a standard thermocycler (i-cycler) and the static microPCR chip. Figure 6 provides a comparison of the efficiencies achieved in the i-cycler and the static microPCR, for various DNA template concentrations used, using as reference intensity (100%) that of the DNA amplified in the i-cycler with a template concentration of 1.25 ng/25 μL PCR reaction. Figure 6 indicates that, first, the static microPCR amplified DNA with a high efficiency compared to that in the standard cycler, and second, the amplification efficiency decreased only slightly (up to 30%) at template concentrations (up to three) orders of magnitude smaller. A *t*-test was performed for comparing statistically the performance of the chip to that of the thermocycler. The *p*-value (for 2 degrees of freedom, two-tail test, unpaired data) was 0.376, therefore the null hypothesis cannot be rejected, i.e., we cannot claim that there was a statistically significant difference between the band intensities in the chip and the thermocycler (for two different template concentrations). Therefore, with the thick PCB microheater chip, highly efficient DNA amplification was demonstrated in the static microPCR, even with low number of DNA template copies (250). This is true not only for short DNA amplicons (117 bp, in this case), but also for longer DNA amplicons, as will be demonstrated below.

**Figure 7:**
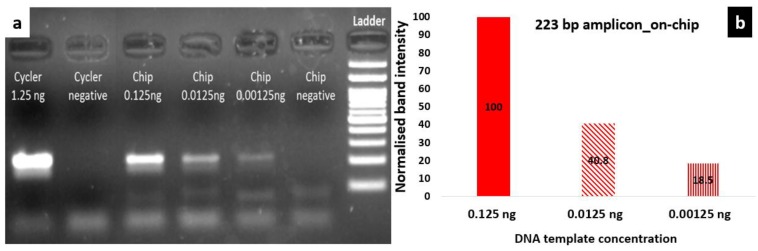
(**a**) Agarose gel electrophoresis image depicting the products (223 bp) from the i-cycler and a static microPCR for purified genomic *Salmonella* DNA using different template concentrations ranging from 0.125 ng to 0.00125 ng per 25 μL PCR reaction (for on-chip amplification). The on-chip experiments were performed simultaneously, in 4 microfluidic chambers of the fluidic chip, in thermal contact with a thick PCB microheater chip; (**b**) comparison of band intensities (normalized to the intensity of PCR with 0.125 ng DNA template) for the on-chip amplification after 40 cycles of 2T PCR employing 3 different DNA template concentrations.

Figure 7a shows agarose gel electrophoresis images depicting the 223 bp products obtained simultaneously from a static four-chamber microPCR for template DNA concentrations ranging from 0.125 to 0.00125 ng per 25 μL PCR reaction, and for 1.25 ng per 25 μL PCR reaction DNA template from the i-cycler, for comparison purposes. Here, DNA amplification was demonstrated in the static microPCR for the 223 bp product, at template concentrations at least as low as 0.00125 ng/25 μL PCR reaction (250 copies). However, in this case, a steeper decrease of the amplification efficiency with the DNA template concentration was demonstrated, attributed possibly to the relative position of the chambers with respect to the chip center. The temperature along the chip is presented in Section 3.1.3 and is shown to decrease from the center to the chip edges, as a result of the heat dissipated from the chip.

#### 3.1.3. Temperature Uniformity on PCB Microheater Chips

Since temperature uniformity is crucial for the efficiency of the PCR reaction, improvement of the temperature uniformity across the microheaters was attempted, aiming at higher amplification efficiency and lower power consumption. Thus, two alterations were made to the design of the microheater: (i) use of a solid copper layer to improve the temperature uniformity across the surface of the chip, and (ii) PCB thickness reduction by half. As shown in Table 2, the thin PCB microheater offered a much better temperature uniformity with a maximum deviation from the set point of 2 °C at the edges, whereas the temperature uniformity of the thick microheater deviated by 4.5 °C at the edges. Regarding the power consumption, the thin microheater seemed to be more energy-demanding, however this was attributed to the larger footprint of the microheater (the surface area of the thin PCB microheater was 1.5-fold larger compared to that of the thick one, so that is compatible with a six-chamber microfluidic chip (Figure 1b)). The effect of the footprint on the power consumption of microPCR devices has been demonstrated through detailed numerical calculations [19]. Despite the fact that the power consumption is slightly larger than those reported in continuous-flow microPCR devices realized in thin polyimide (2.4 W [13]) or PCB (2.7 W [17]), it is far smaller than the power consumption of conventional thermocyclers (which is 500 W or higher [26]). This constitutes the main advantage of the proposed PCB-based miniaturized thermocyclers, combined with their ease of fabrication in large-scale, by leveraging the established and widespread PCB industry.

#### 3.1.4. Thermocycling with a thin PCB Microheater Chip

**Figure 8:**
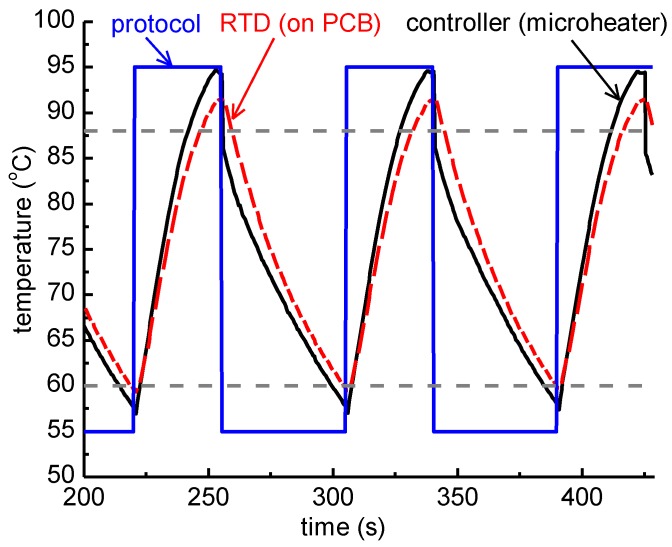
Graphical representation of the temperature measured while running an actual 2T thermocycling experiment with a thin PCB microheater chip. The protocol followed by the temperature controller was 35 s at 95 °C followed by 50 s at 55 °C (blue line). The temperature of the microheaters as obtained from their resistance values acquired by the temperature controller, and the temperature measurement from the RTD placed on the copper layer (see Figure 1) of the microPCR, are also shown. Finally, dashed lines correspond to 88 °C and 60 °C accordingly.

Since the thick PCB microheater chip exhibited quite a large temperature decrease from the center to the edges, as discussed above, attributed to thermal losses from the surface area of the chip, a static microPCR was also developed (on a thin PCB microheater chip) with a copper layer on its surface improving the temperature uniformity. On this PCB microheater, a temperature protocol was implemented similar to the one applied on the thick microheater. In Figure 8, the protocol applied by the temperature controller (35 s at 95 °C and 50 s at 55 °C), the temperature of the microheaters as obtained from their resistance values acquired by the temperature controller, and the temperature as recorded by the RTD, which was in good thermal contact with the copper layer just beneath the PMMA microfluidic chip, are shown. Finally, the temperature range for efficient amplification (>80%), i.e., above 88 °C and below 60 °C, is noted.

**Figure 9:**
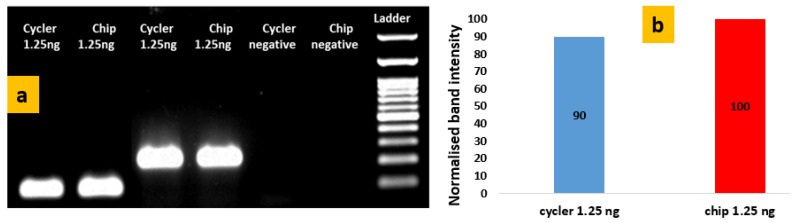
(**a**) Agarose gel electrophoresis image depicting the products (117 bp and 223 bp) from the conventional thermocycler and the static microPCR for purified genomic *Salmonella* DNA using 1.25 ng of DNA/25 μL PCR reaction; (**b**) comparison of normalized band intensities from the thermocycler and the chip after 40 cycles of 2T PCR for the 223 bp amplicon.

DNA amplification experiments were performed in the static microPCR according to the thermal protocol shown in Figure 8. In Figure 9a gel electrophoresis images are shown for DNA (of 1.25 ng of template/25 μL PCR reaction) amplified in both a standard thermocycler (i-cycler) and a static microPCR chip. Figure 9b provides a comparison of the amplification efficiencies achieved in the cycler and static microPCR, for the same DNA template concentration, using as reference intensity (100%) that of the 223 bp amplicon amplified in the static microPCR with a template concentration of 1.25 ng/ 25 μL PCR reaction. The graph indicates similar or even better amplification efficiency for the static microPCR realized on a thin PCB microheater chip. Again, a *t*-test was performed for comparing statistically the performance of the chip to that of the thermocycler, adding to the test described in Section 3.1.2 data from Figure 9, corresponding to different amplicon sizes. The *p*-value (for four degrees of freedom, two-tail test, unpaired data) was 0.573, therefore the null hypothesis cannot be rejected, i.e., we cannot claim that there was a statistically significant difference between the band intensities in the chip and the thermocycler (for two different template concentrations and two amplicon sizes).

The residence time in the denaturation step achieved with the thin T-homogenizing microheater chip (chip with the copper layer) was the same from the RTD and controller measurements. Indeed, Table 3 shows good agreement between the controller and the actual (RTD) denaturation step duration. Regarding the ramping rates, the thin microheater chip exhibited 20% higher cooling rate and 20% slower heating rate. The increase in the cooling rate was attributed to the smaller thermal mass of the thin microheater, which was 0.75 of the mass of the thick one (1.5-times greater footprint area and 2-times lower thickness), and its greater footprint area, which increased the heat losses. The latter also induced a decrease in the heating rate for the thin microheater. It was expected that for thin microPCR devices of the same footprint as the thick one, both cooling and heating rates would be increased (cf. Section 3.2). In order to be further guided on plausible improvements in the operational features of static microPCR implementing PCB microheaters, extensive numerical calculations were carried out, as will be presented in the next section.

### 3.2. Means to Increase the Performance in Terms of Duration and Power Consumption Through Simulations

**Figure 10:**
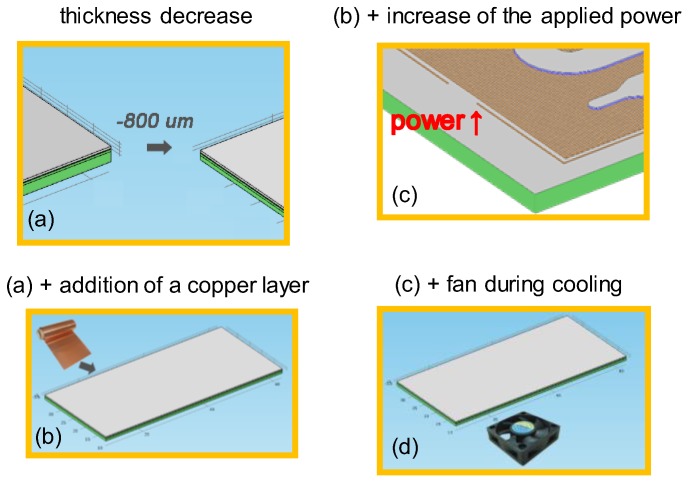
Graphical representations of the means to improve the performance of microPCR device: (**a**) decrease in thickness (case 2); (**b**) (**a**) with the addition of a copper layer between PCB and PMMA layers; (**c**) (**b**) with an increase of the applied power at the resistance (microheater); (**d**) (**c**) with cooling with a fan.

A computational study was performed to investigate means for the improvement of the performance of the microPCR device. In particular, starting from an initial geometry of the microPCR device (case 1), i.e., the design utilized in the first set of experiments, and specific operating conditions, following a 2T-protocol (95–65 °C), several changes were proposed and their effect on the duration, energy consumption, and temperature uniformity in the microPCR device were calculated. In particular, the additive effects of (a) a thinner PCB stack (case 2), (b) a copper layer between the microheaters and the microPCR chamber (case 3), (c) an increase in the power applied to the microheater by the temperature controller (case 4), and (d) implementation of forced cooling (case 5) on the microPCR performance were investigated (Figure 10). The geometry utilized in the computational study is illustrated in Figure 2.

**Figure 11:**
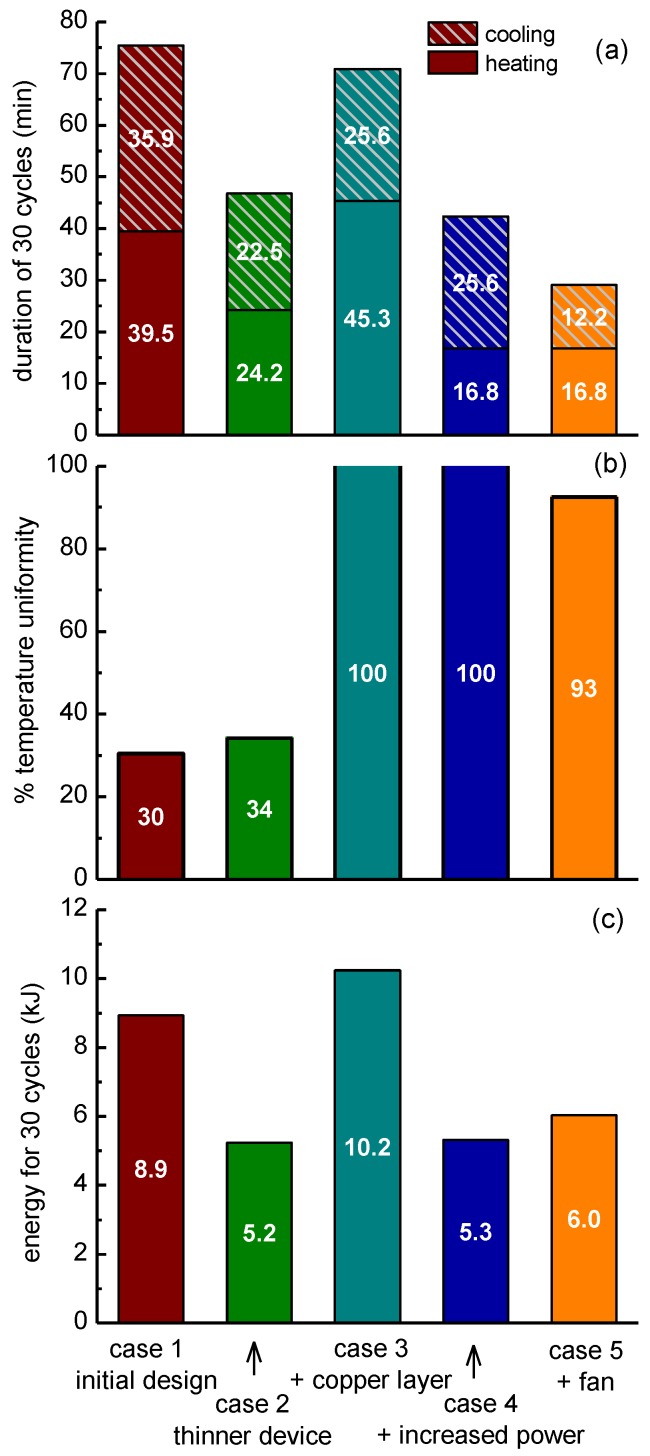
Effects of PCB thickness, addition of copper layer, increased power applied on the microheaters, and active cooling on the microPCR performance, i.e., (**a**) on the duration of a PCR with 30 cycles, (**b**) on the temperature uniformity, and (**c**) on the energy required for a 30-cycle operation, as obtained by numerical calculations.

The transition of the average temperature of the PCR sample (in the chamber of the microPCR device) from 65 °C to 95 °C at each cycle was the result of the power applied to the microheaters, which was in the range of 3.9–4.2 W for case 1. The duration for a 30-cycle PCR protocol was calculated at 75.4 min (Figure 11a). The average heating (cooling) rate was 0.38 °C/s (0.42 °C/s). The temperature uniformity, which is critical for the efficiency of PCR, was quantified by the percentage of the volume of the chamber lying in the acceptable range around the temperature set-points (±2 °C). Hereafter, the temperature uniformity at the annealing-extension step, i.e., around 65 °C, is discussed. The temperature uniformity at the denaturation step, i.e., around 95 °C, was very close to that at the annealing-extension step. For case 1, the temperature uniformity was calculated 30% (Figure 11b). The energy consumption was calculated to be 9.9 kJ for a PCR of 30 cycles (Figure 11c).

Two main problems are observed in the results of Figure 11 for case 1: the PCR duration was long (Figure 11a) and the temperature uniformity was poor (Figure 11b). The next steps were imposed to deal with these problems. The first step to achieve a decrease in the PCR duration and as a consequence of the energy consumption was to decrease the thickness of the stack by 800 μm. The decrease of the thickness decreased the thermal mass and this resulted in a decrease of the PCR duration by 28.7 min (from 75.4 to 46.7 min, Figure 11a); the average heating (cooling) rate increased to 0.62 °C /s (0.67 °C /s). The energy consumption decreased by 3.7 kJ (from ~8.9 to ~5.2 kJ). However, the temperature uniformity was still poor (Figure 11b).

In order to overcome the temperature uniformity problem, a copper layer with thickness of 100 μm was added between the microheaters and the microPCR chamber to homogenize the temperature profile due to the high thermal conductivity of copper. After applying the copper layer, the temperature uniformity reached ~100% (Figure 11b). However, compared to case 1, the PCR duration decreased only 4.5 min (from ~75.4 to ~70.9 min, Figure 11a); the average heating (cooling) rate was 0.33 °C/s (0.59 °C/s). Finally, the energy consumption increased by 1.3 kJ (from 8.9 to 10.2 kJ, Figure 11c).

In order to increase the heating rate, and consequently decrease the PCR duration, the power applied to the microheater was increased to 6.0–6.4 W. The total duration decreased by 33 min (from 75.4 to ~42.4 min, Figure 11a); the average heating (cooling) rate was 0.89 °C/s (0.59 °C/s). The energy consumption decreased by 3.6 kJ (from 8.9 to 5.3 kJ, Figure 11c). The temperature uniformity remained at 100% (Figure 11b). These results can be compared with the experimental results of Section 3.1.4 (microPCR with thin PCB microheater) due to the similarity of the properties of the real device and the simulated one—footprint, material stack, and applied maximum heating power. The calculated total duration (~42.4 min) is in astonishing agreement with the experimental one (85 s/cycle × 30 = 42.5 min). The same is true for the calculated average heating (0.89 °C/s) and cooling rates (0.59 °C/s) in comparison with the experimental ones (0.9 °C/s and 0.6 °C/s, respectively, see Table 3). As far as the energy consumption, the calculated one (5.3 kJ) is in reasonable agreement (within <20%) with the experimental one (1.5 kJ/cycle × 30 cycles = 4.5 kJ, see Table 2).

The transition from 95 °C to 65 °C was performed due to natural cooling in all cases so far. In order to increase the rate of cooling, a fan was added in the configuration. In particular, a commercially available fan was modeled, with a diameter of 4 cm and an air flow of 5 cubic feet per minute (cfm). The addition of the fan did not greatly affect the uniformity (Figure 11b), however, compared to case 1, it decreased the PCR duration by 46.4 min (from 75.4 to 29 min, Figure 11a); the average heating (cooling) rate was 0.89 °C/s (1.23 °C/s). The energy consumption was decreased by 2.9 kJ (from 8.9 to ~6.0 kJ, Figure 11c).

In conclusion, it is evident that a decrease in the thickness of the microPCR PCB stack, an increase in the power applied to the microheaters, the use of active cooling, and the integration of a copper layer between the microheaters and the microPCR chamber can decrease the PCR protocol duration and the energy consumption and increase the temperature uniformity of the device. For example, in the case of a thin PCB, a reduction in the PCR protocol duration from 57 to 39 min can be anticipated (through a projection of the simulation results, for the 40 cycles performed for the experiments presented in Section 3.1.4). Table 4 summarizes the results of the numerical calculations.

Further improvement of the herein introduced PCB-based static microPCR device can be anticipated by incorporating in the PCB, in addition to the microheaters, the microfluidic channels, as it has been demonstrated for continuous-flow microPCR [17]. This would result in a reduction of the thermal mass of the chip and thus of its energy consumption, and is planned to be implemented in the near future, in combination with active cooling, for the next generation of miniaturized PCB thermocyclers.

## 4. Conclusions

A static microPCR was introduced herein, where thermocycling was based on PCB substrates embedding industrially fabricated resistive microheaters. Successful DNA amplification was demonstrated in such devices, with efficiency and total reaction time comparable to that of conventional thermocyclers, nevertheless with a significantly reduced power consumption. The implementation of a thin copper layer on the surface of the PCB, where microfluidic chambers lay, maximized the temperature uniformity and made it possible for multiple reactions to run simultaneously. Numerical calculations demonstrated that the reduction in thickness, the increase in the power applied to the resistive microheater, and the use of active cooling with a fan can reduce the amplification time by more than 60% and the energy consumption by 30%. Therefore, PCB-based microPCR devices hold great potential as miniaturized thermocyclers for reducing cost and power consumption while increasing portability, thus paving the way for PCR adoption in field or point-of-care applications.

## Figures and Tables

**Table 1:** Residence time and ramping rates for the thick printed circuit board (PCB) microheater chip.

55 s at 55°C and 25 s at 90°C	RTD (on PCB)	Controller (at Microheater)
Residence time—denaturation (>88 °C)	4.5 s	3.3 s
Residence time—annealing/extension (<60 °C)	3.7 s	15.5 s
Cooling rate	0.5 °C/s	0.6 °C/s
Heating rate	1.1°C/s	1.4 °C/s

**Table 2:** Temperature profile across the PCB chips and power consumption.

Variable	Thin Microheater (0.8 mm) with Copper Layer		Thick Microheater (1.6 mm)	
temperature at	center	edges	center	edges
denaturation	95 °C	93 °C	95 °C	90.5 °C
annealing/extension	65 °C	63.5 °C	65 °C	62 °C
maximum power	6.3 W		4.9 W	
energy per cycle	150 J		80 J	

**Table 3:** Residence time and ramping rates for the thin PCB microheater chip.

50 s at 55 °C and 35 s at 95 °C	RTD (on PCB)	Controller (at Microheater)
Residence time—denaturation (>88 °C)	13 s	13 s
Residence time—annealing/extension (<60 °C)	4.8 s	6.7 s
Cooling rate	0.6 °C/s	0.7 °C/s
Heating rate	0.9 °C/s	1.2 °C/s

**Table 4:** Summary of the effects of thickness, copper layer, microheater power, and active cooling on the microPCR performance (duration, energy consumed, and temperature uniformity); the values for duration and energy show the % change.

Comparison	Change	Duration	Energy	T-Uniformity
case 2 to 1	case 1 with thickness ↓	38% ↓	41% ↓	34%
case 3 to 1	case 2 + copper layer	6% ↓	15% ↑	100%
case 4 to 1	case 3 + power ↑	44% ↓	41% ↓	100%
case 5 to 1	case 4 + fan	62% ↓	32% ↓	93%
